# Effects of Annealing Temperature on the Microstructure and Mechanical Properties of Asymmetrically Rolled Ultra-Thin Ti-6Al-4V

**DOI:** 10.3390/ma18235436

**Published:** 2025-12-02

**Authors:** Tao Sun, Tan Liu, Mingpei Jiang, Peng Huang, Xianli Yang, Xianlei Hu

**Affiliations:** 1 State Key Laboratory of Digital Steel, Northeastern University, Shenyang 110819, China; suntao@ral.neu.edu.cn (T.S.); jmp1195508455@163.com (M.J.); huangpeng200103@163.com (P.H.); huxl@ral.neu.edu.cn (X.H.); 2 School of Material Science and Engineering, Northeastern University, Shenyang 110819, China; 3 Suzhou Laboratory, Suzhou 215125, China

**Keywords:** asymmetrical rolling, ultra-thin strip, annealing, bipolar plate, TiN coating

## Abstract

In this study, the asymmetrical rolling technique was employed to fabricate 75 μm-thick Ti-6Al-4V ultra-thin strips from the initial 0.45 mm sheet without intermediate annealing, aiming for applications in fuel cell bipolar plates. The rolled strips exhibited good surface quality without cracking. In order to enhance both the mechanical response and the shaping capability of Ti-6Al-4V strips produced by asymmetric rolling, the material was subjected to annealing at various temperatures, and the resulting changes in microstructural features and mechanical performance were systematically examined. The findings indicated that the cold-rolled Ti-6Al-4V exhibited a microstructure primarily composed of subgrains with an average size of approximately 0.41 μm, a feature that contributed to improved corrosion resistance and enhanced ductility after annealing. When the alloy was subjected to heat treatment within the range of 650–800 °C, it was observed that annealing temperatures below 700 °C favored microstructural changes governed predominantly by recovery processes and the onset of recrystallization. At 700 °C, the grains became equiaxed and uniformly distributed, and the dislocation density significantly decreased. The tensile strength reached 887 MPa, while the elongation increased to 13.7%, achieving an excellent strength-ductility balance. Once the annealing temperature rose above 700 °C, noticeable grain growth took place, accompanied by a more pronounced grain-size gradient and a renewed increase in dislocation density. Meanwhile, the dimples observed on the fracture surface became finer, collectively contributing to a decline in tensile elongation. The Ti-6Al-4V ultra-thin strip annealed at 700 °C was used for bipolar plate stamping, producing fine micro-channels with an aspect ratio of 0.43. Finally, TiN coating was applied to the surface, which significantly improved the corrosion resistance and reduced the interfacial contact resistance (ICR), meeting the performance requirements for bipolar plates.

## 1. Introduction

As environmental consciousness continues to rise, Proton Exchange Membrane Fuel Cells (PEMFCs) have emerged as a highly favored substitute for conventional energy systems, largely owing to their superior energy efficiency, absence of carbon emissions, and relatively low operating temperature [1,2]. In the architecture of PEMFCs, bipolar plates constitute a key structural element [3]. They not only ensure mechanical stability but also function as current collectors and distributors, enabling the transfer of electrical energy across individual cells within the stack [4]. In recent years, titanium and its alloys have attracted considerable research interest because of their outstanding strength-to-weight ratio, superior resistance to corrosion, and the comparatively minimal toxicity of titanium ions toward the membrane–electrode assembly [5,6]. Although titanium bipolar plates exhibit superior performance, they still experience slight corrosion under the acidic conditions (pH = 2–5) and high temperatures (70–80 °C) typical in PEMFCs. More importantly, the passivation layer and corrosion products that form on the surface can significantly increase the interfacial contact resistance (ICR), leading to a decrease in conductivity and a loss of cell output efficiency [7]. To mitigate corrosion of titanium substrates and the associated decline in electrical conductivity, surface modification has emerged as the primary and most commonly adopted approach. Among the available treatments, applying corrosion-resistant and electrically conductive protective coatings onto titanium bipolar plates is considered the most practical strategy [8]. Among the various coating options, TiN films provide several notable benefits, such as high hardness, relatively low production cost, superior resistance to corrosion, and favorable electrical conductivity. Moreover, the minimal mismatch in thermal expansion coefficients between TiN and the titanium substrate helps reduce interfacial stresses, thereby enhancing the adhesion strength of the coating [9,10,11].

Bipolar plates contribute roughly 60–80% of the total mass and 30–40% of the overall cost of a PEMFC stack [12,13]. Consequently, advancing the development of ultra-thin bipolar plates is crucial for reducing system expenses. Among the available fabrication techniques, rolling represents a cost-effective and highly efficient process for producing Ti-6Al-4V sheets, and it is responsible for nearly half of all titanium alloy output [14,15]. Hot rolling and warm rolling are commonly employed to produce thicker titanium alloy plates, whereas thin sheets and foils are generally manufactured by cold rolling in industrial practice. Cold rolling has been widely used for processing materials with good plasticity, such as commercially pure titanium [16,17,18,19]. However, for difficult-to-deform materials such as Ti-6Al-4V alloys, the deformation resistance at room temperature is high, and the number of available slip systems for coordinated deformation is limited. As a result, Ti-6Al-4V exhibits poor room-temperature plasticity and severe strain hardening during cold deformation [20,21,22]. Due to its high deformation resistance and limited ductility, conventional cold rolling of Ti-6Al-4V alloys is often restricted [23,24], and the minimum achievable rolling thickness further complicates the production process. Compared with conventional cold rolling, asymmetrical rolling can effectively reduce rolling force and torque [25]. This technique has attracted increasing attention because it allows for improved rolling precision and the possibility of breaking through the minimum rolling thickness limitation [26]. Adjusting factors including the differential speed ratio and deformation conditions enables effective tailoring of the material’s microstructure and mechanical performance [27]. Guo et al. [28] investigated the microstructure and texture evolution of Ti-6Al-4V alloy subjected to asymmetrical rolling followed by recrystallization annealing. Their study revealed that the regions near the roll surface typically experience more pronounced grain refinement during deformation. After annealing, these areas tend to undergo recrystallization first, with recrystallized grains nucleating mainly at the interfaces between coarse grains and substructures, and subsequently growing toward regions with higher dislocation density. By combining asymmetric rolling with static recrystallization, the transverse texture of the material can be effectively weakened, providing a feasible processing route for the industrial production of high-performance rolled titanium alloys.

Implementing severe plastic deformation techniques often results in diminished ductility and can also generate defects, including residual stresses from processing and non-uniform microstructural distributions [29,30,31]. Therefore, subsequent heat treatment is required to modify the internal microstructure of the material and improve its ductility. During the annealing process, the strength of the specimen decreases while the plasticity improves, resulting in an overall enhancement of the comprehensive mechanical properties. Yu et al. [32] produced thin Ti-6Al-4V strips exhibiting high strength and toughness by employing low-temperature rolling followed by brief annealing. Their findings indicated that annealing at 700 °C resulted in a yield strength of 1020 MPa, an ultimate tensile strength of 1126 MPa, and a total elongation of 10.9%. With further increases in annealing temperature, crack and void initiation during tensile loading became more dispersed, allowing the material to accommodate greater deformation and thereby enhancing its ductility.

Existing studies show that the challenges associated with fabricating ultra-thin Ti-6Al-4V sheets have led most research to concentrate on alloys thicker than 0.4 mm, primarily examining their microstructural characteristics and mechanical performance. In contrast, reports addressing the microstructural evolution and mechanical response following substantial asymmetric deformation remain limited. Therefore, in this study, Ti-6Al-4V samples with a total cumulative reduction of 83% were fabricated using asymmetrical rolling technology as the research object. The effects of subsequent heat treatment on the microstructure and mechanical properties were systematically analyzed. Furthermore, the annealed ultra-thin titanium strips were subjected to bipolar plate stamping to evaluate their formability. Finally, a TiN coating was deposited on the Ti-6Al-4V substrate via physical vapor deposition (PVD) to enhance the electrical conductivity and corrosion resistance, providing data support for the application of Ti-6Al-4V ultra-thin strips in fuel cell bipolar plate manufacturing.

## 2. Experimental Methods

### 2.1. Preparation of Experimental Samples

**Figure 1:**
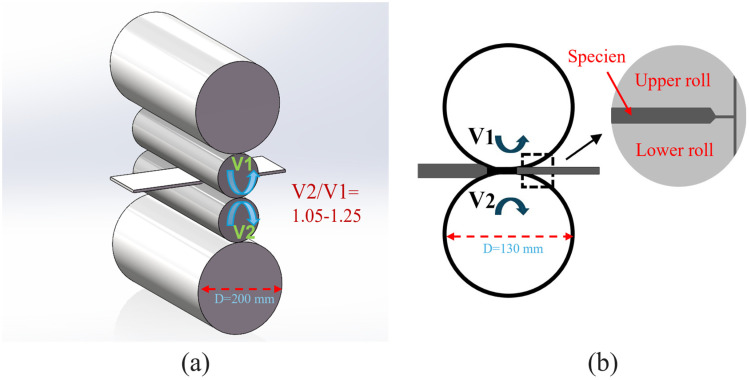
Schematic of asymmetrical rolling of Ti-6Al-4V ultra-thin strips. (**a**) Schematic diagram of the rolling process; (**b**) Schematic diagram of the contact between the rolled sheet and the rolls.

The material used in the experiment was a commercially annealed Ti-6Al-4V plate with a thickness of 0.45 mm, and its chemical composition is provided in Table 1. The rolling experiments were conducted using a four-roll asymmetrical rolling mill independently developed by the National Key Laboratory of Digital Steel at Northeastern University. During rolling, the differential speed ratio was controlled between 1.05 and 1.25, as shown in Table 2. By adjusting the differential speed ratio and related parameters, a shear force was applied along the rolling direction, enabling the Ti-6Al-4V sheet to be rolled down to a thickness of 75 μm, as shown in Figure 1a. During the rolling of the ultra-thin strip, a negative roll gap condition was observed, in which the edges of the upper and lower rolls were tightly pressed together, forming a sealed space, as illustrated in Figure 1b. This condition effectively prevented the lateral flow of the titanium alloy during rolling and thus avoided the formation of edge cracks. Following the rolling process, the Ti-6Al-4V alloy strips with a thickness of 75 μm underwent vacuum annealing at 650 °C, 700 °C, 750 °C, and 800 °C for 1 h to enhance their formability.

### 2.2. Microstructural Characterization and Mechanical Property Testing

Microstructural observations were conducted using a ZEISS GeminiSEM 460 field-emission scanning electron microscope equipped with an EBSD system, manufactured by Carl Zeiss AG, Oberkochen, Germany. The measurements were performed at an accelerating voltage of 20 kV with a step size of 0.1 μm. EBSD datasets were processed and analyzed using Aztec-Crystal 2.1 software. Additionally, the material’s elemental distribution was assessed through energy-dispersive X-ray spectroscopy.

The tensile tests were conducted using a Shimadzu AG-XPLUS 100 kN universal testing machine from Kyoto, Japan. Tensile tests at ambient temperature were carried out on dog-bone specimens measuring 15 mm in gauge length, 6 mm in width, and 0.075 mm in thickness, using a loading rate of 1 mm/min. To assess the suitability of the ultra-thin Ti-6Al-4V strip for bipolar plate manufacturing, forming performance was further examined by conducting stamping experiments on a universal testing machine equipped with a specially designed concave–convex die. The stamped specimens had dimensions of 34 mm (length) × 29 mm (width) × 0.075 mm (thickness). After stamping, the corresponding cross-sections of the microchannels were cut using a wire electrical discharge machining (EDM) system. The samples were then fixed in acrylic cold-mounting resin, and side-profile images of the microchannel regions were captured under an optical microscope. The microchannel dimensions were measured using Image-Pro Plus 6.0 analysis software.

### 2.3. Electrochemical Testing

Electrochemical testing, comprising potentiodynamic polarization measurements (−0.6 V to 2.4 V) and electrochemical impedance spectroscopy, was carried out on Ti-6Al-4V using a CS2350M dual-channel potentiostat/galvanostat manufactured in Wuhan, China. All experiments were performed in a simulated fuel-cell electrolyte composed of H_2_SO_4_ and HF, adjusted to pH 3 and maintained at 70 °C. A standard three-electrode configuration was adopted, employing a saturated calomel electrode as the reference, a platinum electrode as the counter, and a Ti-6Al-4V sample (10 mm × 10 mm) as the working electrode.

### 2.4. ICR Testing

**Figure 2:**
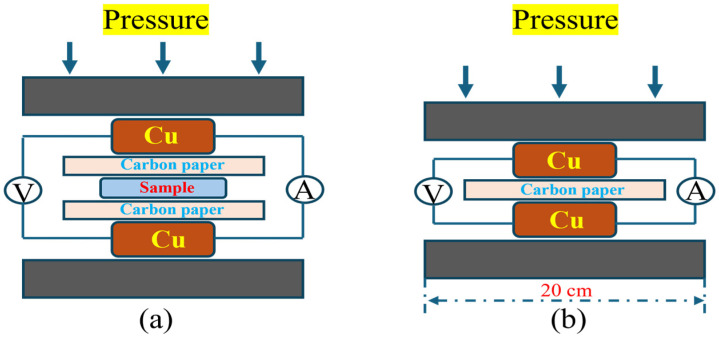
Schematic of the ICR testing principle for Ti-6Al-4V samples. (**a**) Carbon paper and the samples were tested; (**b**) Carbon paper was tested.

In this study, the interfacial contact resistance (ICR) was evaluated following the procedure reported by Wang et al. [33]. During the measurement, the specimen was positioned between two copper plates and layers of carbon paper. Considering that bipolar plates in fuel-cell stacks typically endure a clamping load of 1.3–1.6 MPa, a constant pressure of 1.4 MPa was applied to the copper plates using a Shimadzu AG-XPLUS 100 kN universal testing machine from Japan. First, the total resistance *R*_1_ including the sample was measured, as shown in Figure 2a. Then, the resistance *R*_2_ of the system containing only carbon paper was measured, as also illustrated in Figure 2b. Each sample was tested three times, and the *ICR* of the sample was calculated using the following Equation (1):

(1) $$\mathrm{ICR}=\frac{R_{1}-R_{2}}{2}\cdot S$$

where *S* is the contact area between the titanium alloy sample and the carbon paper.

## 3. Results and Discussion

### 3.1. Microstructure Analysis

**Figure 3:**
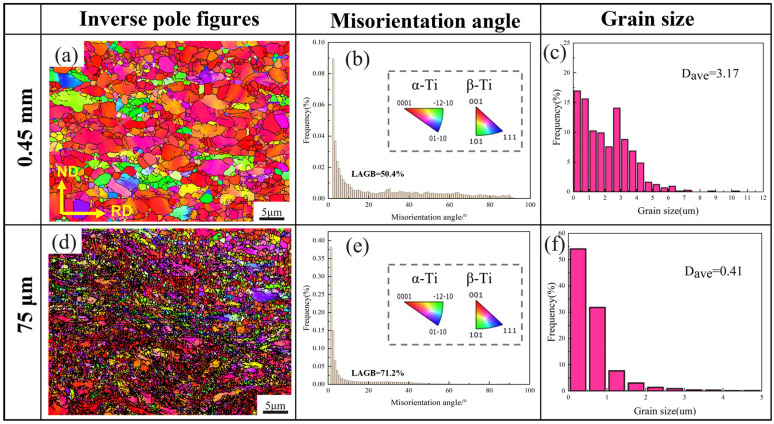
EBSD analysis maps of the original material (OM) and 75 μm cold-rolled Ti-6Al-4V. (**a**,**d**) IPF maps; (**b**,**e**) misorientation angle distribution maps; (**c**,**f**) grain size distribution maps.

Figure 3 presents the EBSD analyses for both the initial Ti-6Al-4V material and the 75 μm cold-rolled sheet. Unlike the elongated grains typically observed after conventional cold rolling, the 75 μm cold-rolled sample, subjected to the shear force during asymmetrical rolling, consists of a microstructure composed of numerous ultrafine grains with slight orientation differences, along with a small amount of elongated α and β phase grains. Figure 3b,e show the distribution of grain-boundary misorientation. Boundaries exhibiting misorientation angles from 2° to 15° are categorized as low-angle grain boundaries (LAGBs), while those with values exceeding 15° are classified as high-angle grain boundaries (HAGBs). The fraction of LAGBs in the OM is 50.4%, and since LAGBs can reflect the level of dislocation density to some extent, this suggests that the OM exhibits a substantial dislocation density. After cold rolling, the proportion of LAGBs in the 75 μm work-hardened specimen increases to 71.2%, accompanied by the formation of numerous sub-grains, implying a high dislocation density. Figure 3c,f illustrate the grain-size profile obtained from the EBSD analysis. After cold rolling, the mean grain size decreases markedly to about 0.41 μm.

**Figure 4:**
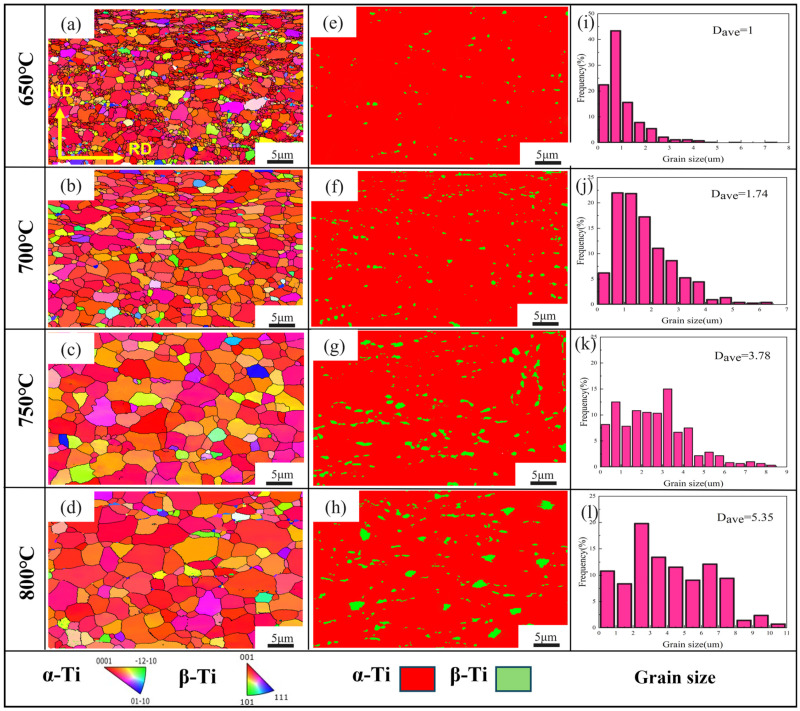
Inverse pole figures (**a**–**d**), phase diagrams (**e**–**h**), and grain size distribution maps (**i**–**l**) of Ti-6Al-4V samples at different annealing temperatures.

Figure 4 presents the EBSD characterization of Ti-6Al-4V samples subjected to 1 h of annealing at various temperatures. In Figure 4a, the specimen treated at 650 °C for 1 h exhibits partial recrystallization, producing a heterogeneous microstructure that includes both coarse and refined α/β grains. At this stage, the mean grain size is roughly 1 μm, and the α phase remains largely untransformed, with only a minor amount of β phase detectable. At an annealing condition of 700 °C for 1 h, the extent of recrystallization shows a significant enhancement. The microstructure becomes fully equiaxed with an average grain size of 1.74 μm, showing a relatively uniform grain size distribution. Meanwhile, the β phase fraction increases and is uniformly distributed around the α phase. As the annealing temperature continues to rise, the grains gradually nucleate, grow, and begin to coarsen. At 800 °C for 1 h, the average grain size increases to 5.35 μm, which should be avoided due to excessive grain coarsening.

**Figure 5:**
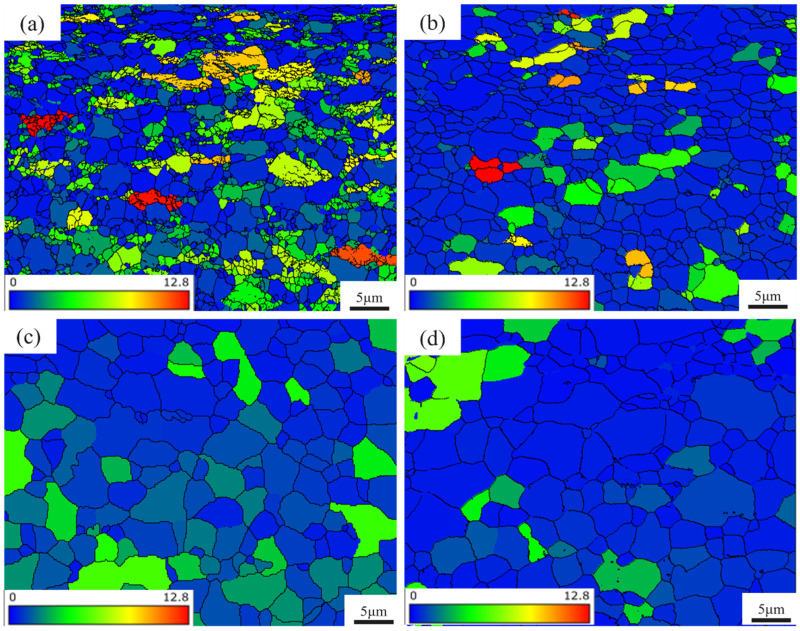
GOS distribution maps of Ti-6Al-4V samples at different annealing temperatures. (**a**) 650 °C; (**b**) 700 °C; (**c**) 750 °C; (**d**) 800 °C.

Figure 5 presents the recrystallization distribution of samples annealed at different temperatures, where blue represents recrystallized grains, red indicates deformed grains, and other colors correspond to substructured grains. As the annealing temperature rises, the proportion of recrystallized regions in the ultra-thin Ti-6Al-4V sheet progressively grows. At 650 °C, the recrystallization driving force is insufficient, leading to a low nucleation rate and only partial recrystallization. Consequently, the microstructure contains a substantial amount of deformed and substructured grains. At 700 °C, the additional thermal input provides greater energy for recrystallization, facilitating the initiation and expansion of new grains, particularly in regions with high stored deformation energy. This occurs because regions with elevated strain energy promote the movement of the original boundaries into the deformed areas, along with the merging of subgrains or their interfaces, ultimately generating distortion-free recrystallized grains [34,35,36]. Comparing Figure 5a,b, it becomes clear that with increasing annealing temperature, the amount of deformed and substructured grains declines, and a larger number of equiaxed, strain-free grains emerge. At 750 °C and 800 °C, recrystallization is nearly finished, and the structure is dominated by recrystallized grains with only a minor portion of substructured zones. Meanwhile, the grains begin to combine and gradually grow in size.

**Figure 6:**
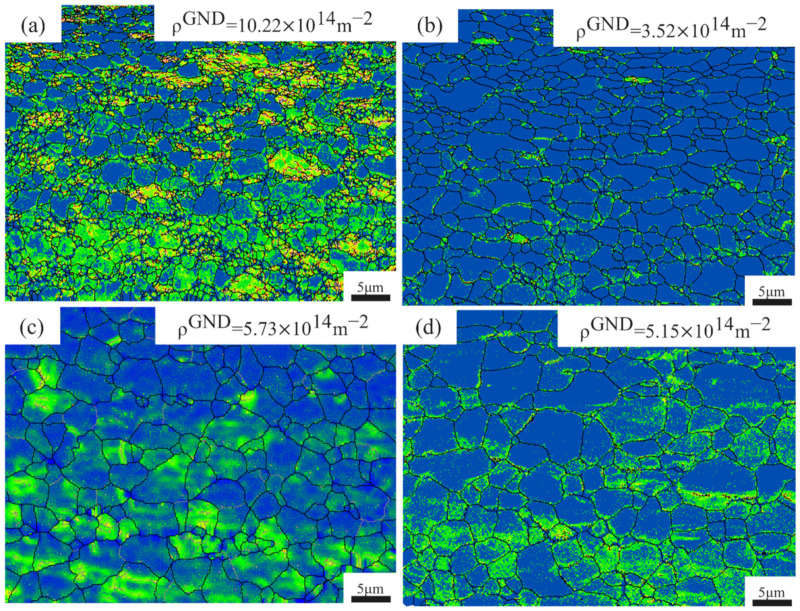
GND distribution maps of Ti-6Al-4V samples at different annealing temperatures. (**a**) 650 °C; (**b**) 700 °C; (**c**) 750 °C; (**d**) 800 °C.

Figure 6 presents the geometrically necessary dislocation (GND) density maps for the ultra-thin Ti-6Al-4V sheets subjected to various annealing temperatures. As seen in the figure, the GND density is relatively high at 650 °C, primarily concentrated around subgrains where recrystallization is limited. At an annealing temperature of 700 °C, the advancement of recrystallization together with the development of equiaxed grains markedly lowers the dislocation density, reaching approximately 3.52 × 10^14^ m^−2^. However, when the heat-treatment temperature rises beyond 700 °C, the GND density increases again, concentrating primarily along the interfaces between coarse and fine grains. Dislocations in some unevenly distributed small grains tend to pile up near the newly formed boundaries [37], resulting in localized zones of elevated dislocation accumulation and higher stored strain energy. This subsequently produces an inhomogeneous grain-size distribution, characterized by a marked contrast between large and small grains. At this stage, the increased dislocation content can also impair the material’s mechanical performance [38].

**Figure 7:**
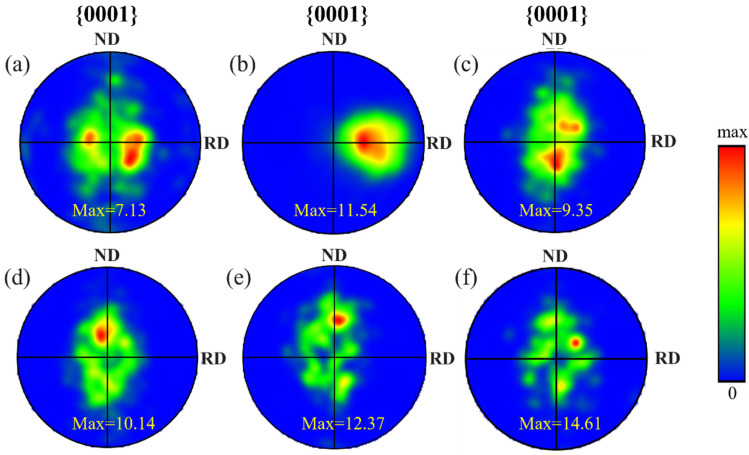
Pole figures of Ti-6Al-4V samples. (**a**) OM; (**b**) 75 μm-CR; (**c**) 75 μm-650 °C; (**d**) 75 μm-700 °C; (**e**) 75 μm-750 °C; (**f**) 75 μm-800 °C.

Figure 7 presents the pole figures of the {0001} plane for Ti-6Al-4V samples in different conditions. As shown in Figure 7a, the original material exhibits a double-peak texture with a relatively low maximum pole density of 7.13. After asymmetrical rolling, severe plastic deformation causes most grains to rotate toward the rolling direction (RD), resulting in a pronounced RD-oriented texture and an increase in pole density. Figure 7c–f illustrate how the pole figures change following annealing at various temperatures. As the annealing temperature rises, the texture intensity initially weakens and subsequently strengthens. After annealing at 650 °C, recovery and partial recrystallization occur within the material, where subgrains merge and develop into new grains, leading to more random orientations and a reduction in texture intensity. At 700–800 °C, as recrystallization becomes nearly complete and grain growth proceeds, certain grains with low-energy boundaries or favorable orientations grow preferentially, resulting in a renewed enhancement of the texture intensity.

### 3.2. Mechanical Properties Analysis

**Figure 8:**
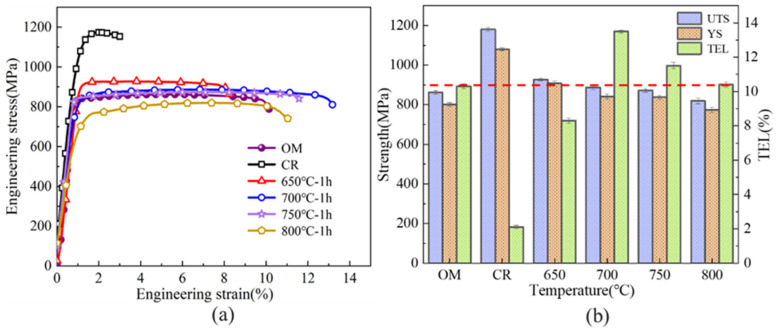
Mechanical properties of Ti-6Al-4V in the cold-rolled state and after annealing at different temperatures. (**a**) Engineering stress–strain curves; (**b**) Bar chart of mechanical property variations.

Mechanical property data for the unprocessed Ti-6Al-4V sheet, the 75 μm work-hardened sample, and the annealed specimens are displayed in Figure 8. From Figure 8a, it is evident that the work-hardened Ti-6Al-4V attains the highest strength values, with UTS and YS measured at 1180 MPa and 1080 MPa. Due to its high dislocation density. However, the total elongation (TEL) is relatively low, at only 3.0%. With increasing annealing temperature, the material undergoes softening, and both UTS and YS gradually decrease. After annealing at 650 °C for 1 h, the UTS and YS decrease to 927 MPa and 907 MPa, while the total elongation increases to 8.3%. Since the recrystallization fraction at this temperature remains relatively low and a high proportion of dislocations still exist, the elongation does not improve significantly. Upon annealing at 700 °C, the extent of recrystallization grows, leading to a pronounced decrease in dislocation density. The formation of equiaxed grains promotes dislocation motion and facilitates plastic deformation [39], leading to an elongation of 13.7%, which represents the optimal combination of strength and ductility. When the annealing temperature is elevated to 750 °C and 800 °C, the proportion of recrystallized grains increases further while lattice distortion is alleviated, resulting in an additional decline in strength. At the same time, the grains begin to grow and coarsen. According to Yan et al. [40], enlargement of the α phase during tensile deformation reduces both the tensile strength and the elongation of Ti-6Al-4V specimens.

Figure 8b displays a bar chart that compares the mechanical properties of Ti-6Al-4V under various processing conditions. The data clearly show that the 75 μm cold-rolled specimen attains the greatest tensile strength while demonstrating the lowest ductility. As the annealing temperature rises, the strength of the Ti-6Al-4V samples decreases, while the elongation initially increases and then declines. The dashed red curve in Figure 8b denotes the elongation of the untreated Ti-6Al-4V sheet. It is clear that after annealing at 700–800 °C, the elongation increases markedly and surpasses that of the original material. This enhancement results from the substantial stored strain energy and the fine grains generated during severe deformation, which facilitate complete recrystallization during the annealing process. According to the Hall-Petch relationship, finer grains not only enhance the strength but also enable a more uniform distribution of slip systems during deformation, thereby improving the overall elongation of the material.

**Figure 9:**
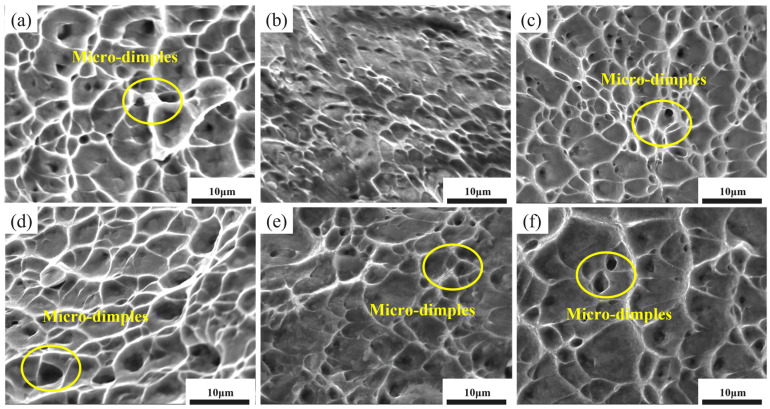
SEM images of the tensile fracture surfaces of Ti-6Al-4V samples. (**a**) OM; (**b**) 75 μm-CR; (**c**) 75 μm-650 °C; (**d**) 75 μm-700 °C; (**e**) 75 μm-750 °C; (**f**) 75 μm-800 °C.

Figure 9 presents SEM images displaying the tensile fracture morphologies of the various Ti-6Al-4V samples. In Figure 9a, the fracture surface of the original material is characterized predominantly by equiaxed dimples, indicative of a classic ductile fracture mode. In contrast, the 75 μm cold-rolled sample shows almost no micro-dimples, with distinct tearing ridges and layered fracture features, indicating poor plasticity. Figure 9c–f display the fracture characteristics of the 75 μm ultra-thin sheets subjected to various annealing temperatures. As the annealing temperature rises, the dimples enlarge initially and then diminish in size, a pattern that aligns with the observed changes in elongation. Following annealing at 650 °C, the fracture surface is dominated by small and shallow equiaxed dimples, suggesting that the plasticity begins to recover. At 700 °C, the dimples become larger and deeper, and are uniformly distributed, indicating the highest elongation and best ductility. As the annealing temperature rises further to 750 °C and 800 °C, the dimples become smaller and fewer in number, corresponding to the observed decrease in elongation.

### 3.3. Ti-6Al-4V Bipolar Plate Forming Analysis

**Figure 10:**
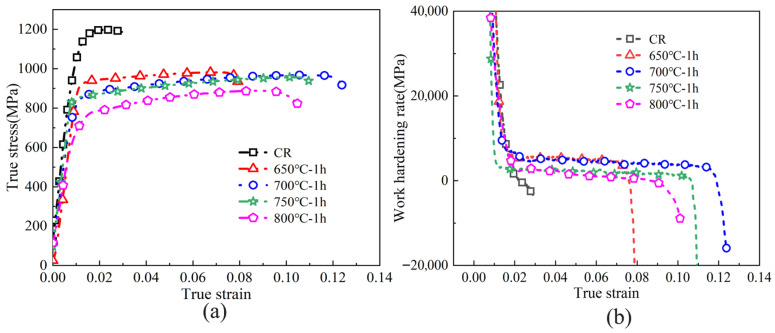
True stress–strain and work. (**a**) True stress–strain curves; (**b**) Work hardening rate curves.

There are various forming methods for metallic bipolar plates, such as electrical discharge machining (EDM) [41], electrochemical machining (ECM) [42], vacuum die casting [43], and additive manufacturing [44,45]. However, considering both cost and large-scale production requirements, plastic forming processes are regarded as the most suitable methods for fabricating metallic bipolar plates [46], including stamping and related techniques [47,48]. Before performing the stamping process, the work-hardening behavior of the annealed material must be assessed. Figure 10 displays the true stress–strain curves along with the corresponding work-hardening rate curves for samples annealed at various temperatures. The work-hardening rate (*θ*) is determined according to the following equation:

(2) θ=dσ/dε

where *θ* is the work-hardening rate, *σ* is the true stress, and *ε* is the true strain.

Figure 10b reveals that the work-hardening behavior and the span of the uniform deformation stage respond differently to changes in annealing temperature. The cold-rolled sample, which contains a high dislocation density, experiences severe dislocation entanglement and saturation. Consequently, the work-hardening rate drops sharply as the true strain increases, resulting in a limited forming capability and weak resistance to deformation. After annealing Ti-6Al-4V at different temperatures, it was observed that with elevated annealing temperatures, the work-hardening rate and the extent of the uniform deformation region show an initial rise followed by a reduction. At 650 °C, the work hardening rate was relatively high, but the uniform deformation stage was short, indicating a reduced ability of Ti-6Al-4V to resist strain instability during plastic deformation. At an annealing temperature of 700 °C, the sample showed the maximum work-hardening rate and the most extended uniform deformation stage. In contrast, upon raising the temperature further to 750 °C and 800 °C, both the work hardening rate and the length of the uniform deformation stage decreased. Based on the above analysis, samples annealed at 700–800 °C were selected for bipolar plate stamping.

**Figure 11:**
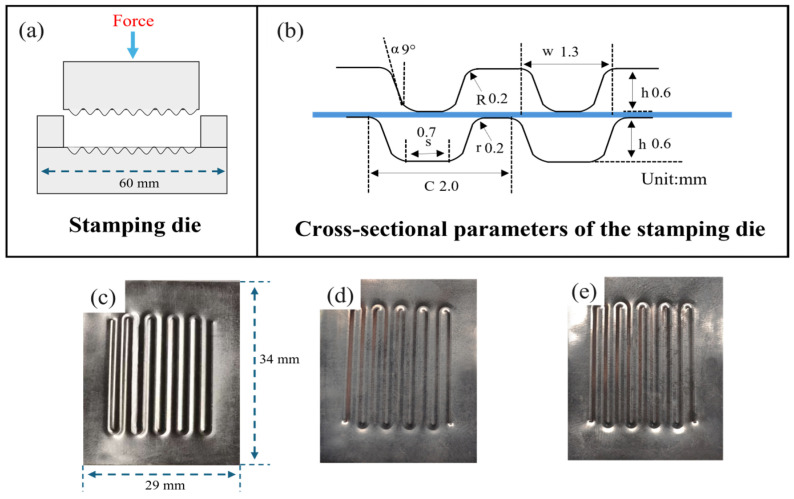
Stamping die and stamped samples. (**a**) Schematic diagram of stamping; (**b**) Cross-sectional parameters of the stamping die; (**c**–**e**) Formed samples annealed at 700–800 °C.

The cross-sectional parameters of the stamping die used in this experiment are shown in Figure 11. Channel geometry is characterized by several parameters, including depth (h), width (w), outer and inner corner radii (R and r), draft angle (α), and pitch (c). The aspect ratio (*Ar*), calculated using Equation (3), is an important metric for evaluating stamping behavior, where h denotes the depth and w the width of the channel.

(3) Ar=h/w

where *h* corresponds to the depth of the channel, while *w* refers to its width.

**Figure 12:**
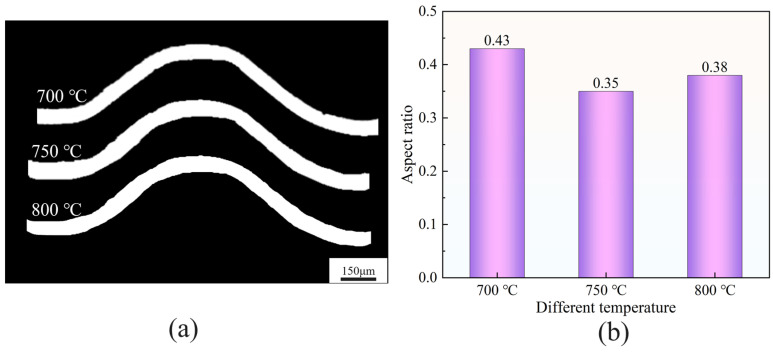
(**a**) Microchannel thickness distribution; (**b**) Aspect ratio values.

Figure 11c–e show the bipolar plate samples formed by stamping Ti-6Al-4V ultra-thin strips annealed at different temperatures ranging from 700 to 800 °C, and Figure 12a presents the corresponding microchannel micrographs. When the bipolar plates were stamped using the die designed with the above cross-sectional parameters, it was observed that, compared with the samples stamped after annealing at 750 °C and 800 °C, the microchannels formed after annealing at 700 °C exhibited the best morphology, with a depth-to-width ratio reaching 0.43. Therefore, the samples annealed at 700 °C were selected for subsequent studies on potentiodynamic corrosion and interfacial contact resistance.

## 4. Electrochemical Corrosion Behavior Test

**Figure 13:**
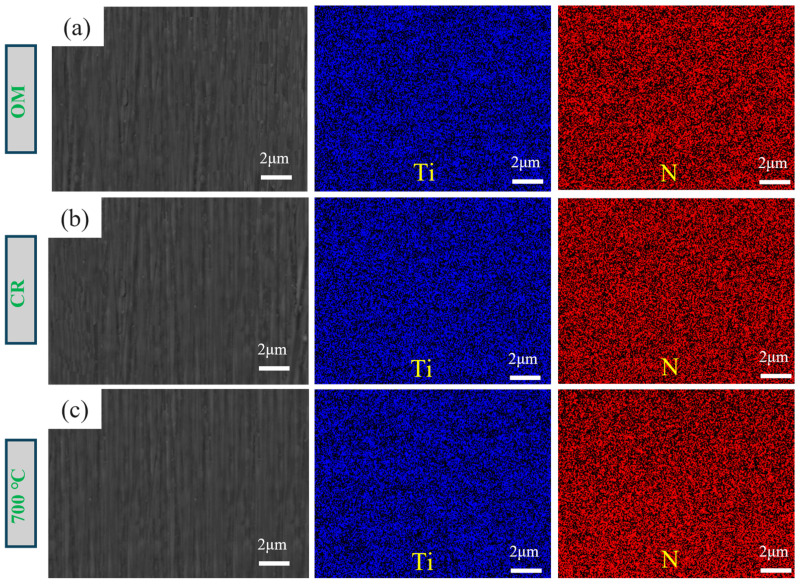
SEM images showing the TiN coating and elemental distribution. (**a**) OM; (**b**) 75 μm-CR; (**c**) 75 μm-700 °C.

To strengthen the alloy’s resistance to electrochemical corrosion and optimize its electrical conductivity, a PVD coating treatment was applied to its surface. The 0.45 mm OM sample, the 75 μm cold-rolled sample, and the sample annealed at 700 °C were coated with TiN. The surface morphology and elemental distribution are shown in Figure 13, where the Ti and N elements are uniformly distributed.

**Figure 14:**
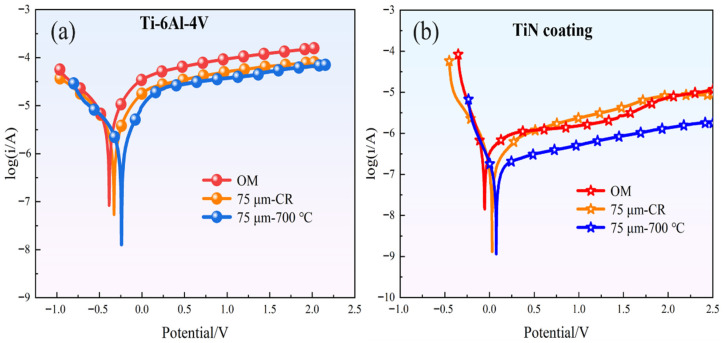
Potentiodynamic polarization curves. (**a**) Ti-6Al-4V substrate; (**b**) TiN coating.

Figure 14 shows the potentiodynamic polarization curves of the Ti-6Al-4V substrate and the coated samples, and the corresponding values are listed in Table 3. Figure 14a shows the polarization curve of the Ti-6Al-4V substrate. Compared with the as-received Ti-6Al-4V, the cold-rolled sample exhibits an increase in E_corr_ and a decrease in I_corr_. Generally, a higher E_corr_ indicates a lower corrosion tendency, while a lower I_corr_ corresponds to a slower corrosion rate. This improvement is attributed to the formation of ultrafine grains and the enhanced {0001} texture after asymmetrical rolling, as ultrafine-grained titanium exhibits faster passivation and better corrosion resistance than coarse-grained titanium [49]. Figure 14b shows the polarization curves after TiN coating. After coating, both E_corr_ and I_corr_ change significantly, indicating a substantial improvement in corrosion resistance. Notably, the 75 μm-700 °C coated sample exhibits an I_corr_ of 9.865 × 10^−8^ A/cm^2^, meeting the corrosion resistance requirements for fuel cell bipolar plates.

**Figure 15:**
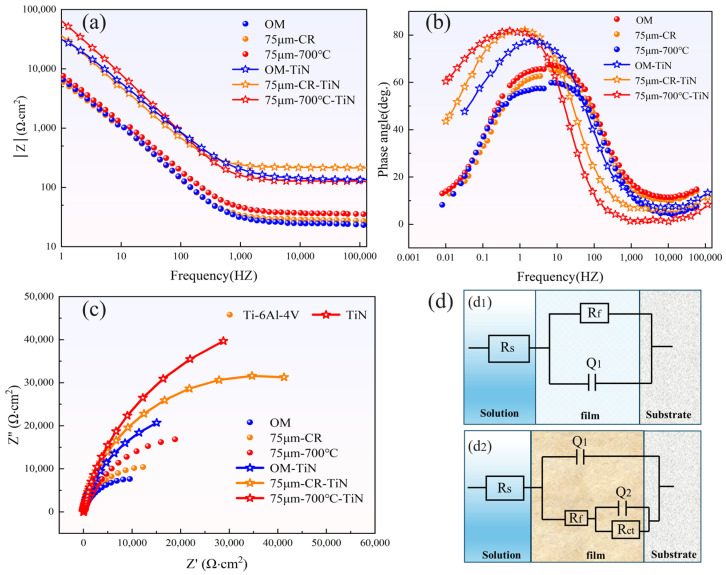
Impedance spectra and equivalent circuit fitting of Ti-6Al-4V. (**a**) Bode modulus; (**b**) Bode phase angle; (**c**) Nyquist diagram; (**d**) equivalent circuits, where (**d1**) represents the Ti-6Al-4V substrate and (**d2**) represents the TiN coating.

Figure 15 presents the impedance spectra and the corresponding equivalent circuit fitting results for the Ti-6Al-4V substrate and the TiN-coated samples. Figure 15a shows the Bode magnitude plot. As illustrated, the impedance modulus of the coated sample at low frequencies is significantly higher than that of the Ti-6Al-4V substrate, indicating that the TiN coating markedly increases the polarization resistance of the material. Figure 15b displays the Bode phase plot. The coated sample exhibits a broad peak with a phase angle close to 80°, suggesting pronounced capacitive behavior and enhanced electrochemical stability introduced by the TiN coating. Figure 15c shows the Nyquist plots of the substrate and coated samples. A larger semicircle radius corresponds to a higher charge transfer resistance and thus better corrosion resistance. The noticeable increase in the semicircle radius after coating further confirms the improved electrochemical stability provided by the TiN layer.

Figure 15d shows the equivalent circuit models obtained after fitting the impedance curves. The fitting values are shown in Table 4. Two circuit models were proposed: model d1 is a single-time-constant equivalent circuit for the Ti-6Al-4V substrate, while model d2 is a two-time-constant equivalent circuit for the TiN-coated sample. In the circuits, Rs represents the solution resistance, Rf represents the resistance of the passive film, Rct denotes the charge transfer resistance, and Q corresponds to the constant phase element.

## 5. ICR Testing

**Figure 16:**
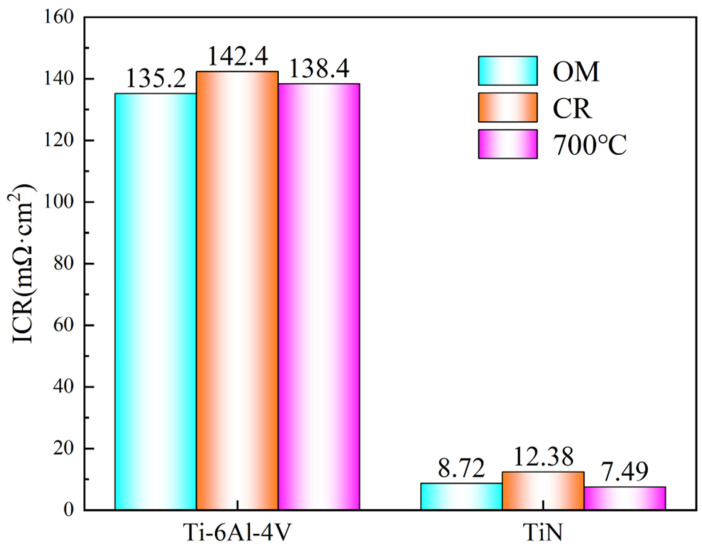
ICR values of Ti-6Al-4V samples in different conditions.

Figure 16 illustrates that the uncoated Ti-6Al-4V samples possess comparatively high ICR values, which stem from the formation of a compact TiO_2_ passive layer on their surfaces. This oxide film results in inadequate electrical conductivity of the Ti-6Al-4V substrate, rendering it unsuitable for use as bipolar plates [50]. It is also observed that the ICR slightly increases after rolling, which is opposite to the trend observed for I_corr_. This is likely due to the formation of subgrains and an increased number of grain boundaries after rolling, which promote a denser passive film. After TiN coating, the ICR values of all three sample conditions are significantly reduced, with the TiN-OM and TiN-700 °C samples achieving ICR values below 8.72 mΩ·cm^2^, meeting the U.S. 2025 DOE standard.

## 6. Conclusions

In this study, the asymmetrical rolling technique was applied to Ti-6Al-4V alloy to produce ultra-thin strips for fuel cell bipolar plates. Without intermediate annealing, the initial 0.45 mm-thick Ti-6Al-4V sheet was rolled down to 75 μm. The microstructural and mechanical responses of the ultra-thin strips were examined after annealing at temperatures ranging from 650 to 800 °C. Following this assessment, the annealed strips underwent stamping tests to determine their suitability for forming bipolar plates. In the final stage, a PVD coating was introduced to further improve both the corrosion resistance and electrical conductivity of the Ti-6Al-4V ultra-thin strips. The key findings are summarized below:

(1) After large plastic deformation through asymmetrical rolling, the Ti-6Al-4V samples exhibited microstructures composed of numerous substructured grains formed under shear stress. Significant grain refinement was achieved, which is beneficial for improving both corrosion resistance and elongation.
(2) With increasing annealing temperature, the grain size gradually increased, and the GND density first increased and then decreased. At 700 °C, recrystallization was nearly complete, with an average grain size of 1.74 μm and a GND density reduced to 3.52 × 10^14^ m^−2^. When the annealing temperature exceeded 700 °C, the grains coarsened, the α and β phase size gradients increased, and the GND density rose again, resulting in a decrease in elongation.
(3) As the annealing temperature was raised, a gradual decrease in the tensile strength of the Ti-6Al-4V ultra-thin strip was observed, while the elongation first increased and then declined. At 700 °C, uniformly equiaxed grains were formed, whose boundaries promoted dislocation movement and enhanced strain coordination, achieving an optimal balance of strength (887 MPa) and ductility (13.7%).
(4) Fractography analysis showed that the dimple morphology varied with annealing temperature. At 700 °C, large and deep dimples were observed, indicating typical ductile fracture. Moreover, the work-hardening rate curve exhibited the longest uniform deformation stage, which helps prevent strain localization during stamping.
(5) After TiN coating, the Ti-6Al-4V ultra-thin strip exhibited significantly improved corrosion resistance and electrical conductivity. The impedance spectra and fitting results clearly show that the corrosion resistance and stability of the material are significantly improved after coating. In particular, the sample annealed at 700 °C showed a decreased corrosion current density of 9.865 × 10^−8^A·cm^−2^ and ICR of 7.49 mΩ·cm^2^, meeting the 2025 DOE standard.

## Figures and Tables

**Table 1:** Chemical composition of the initial Ti-6Al-4V sheet (mass fraction, %).

Ti	Al	V	Fe	C	N	O
Bal	6.30	4.19	0.191	0.010	0.004	0.124

**Table 2:** Asymmetrical rolling process parameters.

Pass	Thickness Before Rolling (H)/μm	Thickness After Rolling (h)/μm	Thickness After Rolling γ/%	Asymmetrical Speed Ratio (i)
1	450	350	22.2	1.05
2	350	290	17.1	1.05
3	290	246	15.2	1.05
4	246	211	14.2	1.05
5	211	183	13.3	1.07
6	183	160	12.6	1.07
7	160	143	10.6	1.07
8	143	129	9.8	1.10
9	129	117	9.3	1.10
10	117	107	8.5	1.13
11	107	100	6.5	1.15
12	100	94	6.0	1.15
13	94	89	5.5	1.17
14	89	84	5.5	1.23
15	84	80	5.0	1.23
16	80	77	3.8	1.25
17	77	75	2.6	1.25

**Table 3:** **E_corr_** and **I_corr_** of Ti-6Al-4V sheets in different states.

Sample	E_ccor_/V	I_ccor_/A·cm^−2^	β_a_/mv	β_c_/mv
OM	−0.389	5.207 × 10^−6^	181.3	221.2
75 μm-CR	−0.33	2.366 × 10^−6^	185.4	202.1
75 μm-700 °C	−0.237	1.302 × 10^−6^	191.7	227
OM-TiN	−0.059	7.277 × 10^−7^	348.9	138.8
75 μm-TiN	0.029	3.211 × 10^−7^	326.7	135.9
75 μm-700 °C-TiN	0.077	9.865 × 10^−8^	310.4	143.7

**Table 4:** Fitting parameters of the equivalent circuits for Ti-6Al-4V sheets in different conditions.

Sample	R_s_/Ω·cm^2^	R_f_/Ω·cm^2^	R_ct_/Ω·cm^2^	Q_1_/F·cm^−2^	Q_2_/F·cm^−2^
OM	30.68	6.93 × 10^3^	-	4.72 × 10^−4^	-
75 μm-CR	29.73	8.65 × 10^3^	-	3.15 × 10^−4^	-
75 μm-700 °C	28.35	9.36 × 10^3^	-	2.41 × 10^−4^	-
OM-TiN	25.18	2.28 × 10^4^	5.72 × 10^4^	4.11 × 10^−4^	2.87 × 10^−4^
75 μm-CR-TiN	27.43	4.36 × 10^4^	6.17 × 10^4^	2.53 × 10^−4^	1.55 × 10^−4^
75 μm-700 °C-TiN	24.27	7.28 × 10^4^	8.28 × 10^4^	2.24 × 10^−4^	1.01 × 10^−4^

## Data Availability

The original contributions presented in this study are included in the article. Further inquiries can be directed to the corresponding authors.
